# Supplementary material for: Diarrhea and related personal characteristics among Japanese university students studying abroad in intermediate- and low-risk countries
Source: PLoS One. 2023 Feb 24;18(2):e0279426. doi: 10.1371/journal.pone.0279426 (PMC9956663; doi:10.1371/journal.pone.0279426)
Supplement: S1 Table — (DOC) [file pone.0279426.s001.doc]

**S1 Table. Sensitivity analyses of personal characteristics associated with diarrhea, by the destination’s risk categories ^a^**

|  | **Intermediate-risk countries ^a^** | **Low-risk countries ^a^** |
| --- | --- | --- |
|  | **aOR ^b^ (95% CI)** | **aOR ^b^ (95% CI)** |
| **First episode during the 1st week** | | |
| Age group |  | |
| 10s (vs. 20s) | 1.97 (0.79, 4.92) | 1.26 (0.51, 3.09) |
| Academic year |  | |
| 2017 (vs. 2016) | 1.21 (0.54, 2.73) | 4.45 (1.4, 14.15) |
| 2018 (vs. 2016) | 0.78 (0.34, 1.79) | 1.84 (0.49, 6.85) |
| Sex |  | |
| Male (vs. female) | 1.71 (0.88, 3.34) | 0.59 (0.21, 1.66) |
| Overseas travel experience |  | |
| Yes ^c^ (vs. no) | 1.71 (0.87, 3.39) | 0.81 (0.34, 1.95) |
| **Extending at-risk period by 2 days** | | |
| Age group |  | |
| 10s (vs. 20s) | 2.79 (1.25, 6.24) | 1.31 (0.59, 2.95) |
| Academic year |  | |
| 2017 (vs. 2016) | 1.46 (0.73, 2.93) | 3.59 (1.34, 9.61) |
| 2018 (vs. 2016) | 0.93 (0.46, 1.85) | 1.61 (0.53, 4.90) |
| Sex |  | |
| Male (vs. female) | 1.97 (1.12, 3.46) | 0.65 (0.27, 1.59) |
| Overseas travel experience |  | |
| Yes ^c^ (vs. no) | 2.19 (1.23, 3.9) | 1.05 (0.47, 2.35) |
| **Excluding Singapore from intermediate-risk countries (n=135)** | | |
| Age group |  | |
| 10s (vs. 20s) | 2.24 (0.80, 6.29) |  |
| Academic year |  | |
| 2017 (vs. 2016) | 1.11 (0.42, 2.90) |  |
| 2018 (vs. 2016) | 0.95 (0.36, 2.48) |  |
| Sex |  | |
| Male (vs. female) | 1.69 (0.81, 3.54) |  |
| Overseas travel experience |  | |
| Yes ^c^ (vs. no) | 1.95 (0.90, 4.22) |  |

Abbreviations: n, number; aOR, adjusted odds ratio; CI, confidence interval

^a^ Intermediate-risk countries included Malaysia, Singapore, Thailand, and China; and low-risk countries included Australia, USA, France, UK, Ireland, Canada, and South Korea.

^b^ Age group, academic year, sex, and overseas travel experience were simultaneously adjusted for.

^c^ Two overseas travel experience categories (ever or never visited the destination) were combined.
