# Supplementary material for: Diarrhea and related personal characteristics among Japanese university students studying abroad in intermediate- and low-risk countries
Source: PLoS One. 2023 Feb 24;18(2):e0279426. doi: 10.1371/journal.pone.0279426 (PMC9956663; doi:10.1371/journal.pone.0279426)
Supplement: Attachment — Submitted filename: Response to Reviewers.docx [file pone.0279426.s002.docx]

October 4, 2022

Dr. Alice Coles-Aldridge

Editorial Office

*PLOS ONE*

Dear Dr. Aldridge,

On behalf of the authors, I am pleased to submit our revised manuscript entitled “Diarrhea and related personal characteristics among Japanese university students studying abroad in intermediate- and low-risk countries” for possible publication as an original article in *PLOS ONE*. The revised manuscript consists of 225 words for abstract 2,489 words for main text, 4 tables, and 1 online material (1 supporting information file).

Following the reviewer’s comments, we carefully revised our manuscript, and our point-by-point responses to the comments are attached.

Responses to Academic Editor

We thank the academic editor for this opportunity to revise our manuscript. We carefully revised the manuscript following the reviewer #1’s comments. First, we added the sentences in the limitation of Discussion section that because this study was conducted in the spring or summer, seasonal component may in part account for the risk-based findings in non-tropic countries. Next, to lower the risk of diarrhea in teenagers, males, and those with overseas travel experience, who were suggested to be high risk, we added public health messages for the groups in the Conclusion section. In addition, we modified the manuscript following the journal style requirements, provided additional information regarding participant consent and funding statement, and included captions for our supporting information file. As a result of this revision, we believe that our manuscript has greatly improved.

Responses to comments from Reviewer #1

*A very nice paper on TD among students studying abroad. A very enjoyable read, robustly analyzed, and very clearly written.*

We thank the reviewer for his/her constructive comments and carefully revised the manuscript following the comments.

*1. Vibrio cholerae should have the species name lowercase.*

Response:

Thank you for your pointing out. We corrected the species name in lower case as follows:

“Considering the immunity against norovirus [14] and *Vibrio cholerae* [15], ABO blood group was dichotomized as ‘O’ or other types including unknown blood type.” (page 5, line 83)

*2. For this statement in the discussion, I think the authors mean to adjust their plans: "Another possible reason is that a longer period of stay made it easier to adjust the schedules."*

Response:

Thank you for your pointing out. We replaced the word “schedules” to “plans” in the sentence as follows:

“Another possible reason is that a longer period of stay made it easier to adjust their plans.” (page 13, lines 217 to 218)

*3. In the limitations and the discussion paragraphs the authors also need to note that this study was done in the spring/summer and there may be a component of seasonality among non-tropics (low risk) destinations that may account for some of their risk-based findings.*

Response:

We agree. While acknowledging the potential impact of seasonal component on the risk-based findings in non-tropic (low-risk) countries, we thus added the sentences in the limitation of Discussion section as follows:

“Fourth, this study was conducted in the spring or summer. The seasonal component may in part account for the results based on the diarrhea risk in non-tropic (low-risk) countries.” (page 14, lines 259 to 261)

*Results*

*4. The conclusions should be broadened. What exactly do the authors think needs to happen for these groups to LOWER their risk? In other words, what is the public health message to these groups? Simply restating results in the conclusion isn't very helpful here.*

Response:

We agree. To lower the risk of diarrhea in teenagers, males, and those with overseas travel experiences, who were suggested to be high risk, public health messages for the groups are necessary. We thus modified the sentences in the Conclusion section as follows:

“Diarrhea was a common illness among Japanese university students studying abroad, which is comparable with general travelers, e.g., tourists. Younger students, males and those with overseas travel experience need to be cautious about the risk of diarrhea, specifically in intermediate-risk countries. To lower the risk, they should avoid high risk behaviors, e.g., through careful choices of safe food and beverages.” (page 15, lines 268 to 272)

*5. In the methods the authors need to state how data was managed (i.e., what software was used): SAS? R? Statistical packages should also be mentioned.*

Response:

Thank you for your pointing out. We added the information about the statistical package we used as follows:

“Stata SE statistical software (version 16.1; StataCorp, College Station, TX, USA) was used for all the analyses.” (page 7, lines 129 to 130)

We believe that our paper has improved as a result of the reviewer’s helpful suggestions. We thank you for the opportunity of re-submission. We hope that, with these modifications, our paper can now be accepted for publication. We look forward to hearing from you.

Sincerely yours,

Michiyo Yamakawa, PhD, on behalf of all authors
